# Cases of intervention refusal encountered by public health nurses in Japan and characteristics of their support– qualitative analysis of described mother-child and elderly cases

**DOI:** 10.1186/s12912-021-00706-z

**Published:** 2022-02-03

**Authors:** Reiko Okamoto, Misaki Kiya, Keiko Koide, Miho Tanaka, Masako Kageyama

**Affiliations:** Division of Health Sciences, Osaka University Graduate School of Medicine, Yamadaoka 1-7, Suita-city, Osaka, 565-0871 Japan

**Keywords:** Intervention refusal, Public health nurse, Mother-child case, Elderly case

## Abstract

**Background:**

The purpose of this study is to clarify the actual situation of the cases and the characteristics of support, focusing on mothers and their children, and elderly persons among the cases of intervention refusal encountered by public health nurses (PHNs) in Japan.

**Methods:**

The data were descriptions of intervention refusal cases that were freely described by PHNs working for prefectural and municipal governments in questionnaire surveys nationwide. The characteristics of the cases and the support were categorized according to the situation of the case, and the number of descriptions was summarized and interpreted.

**Results:**

The results revealed that interventions involving mothers and children were refused in most of by mother or parents. The refusals were related to child abuse, parental mental illness, obsessiveness, and complex backgrounds. The actual status of intervention refusal in elderly persons, interventions are frequently refused by elderly persons themselves in the case of self-neglect and by family members living with the elderly in the case of abuse. The refusals were related to mental disorders or dementia and living alone. In both cases, PHNs provided support in collaboration with multi-disciplinary and multi-agencies, and attempts were made to alleviate the situation of refusal to intervene, from detecting cases through contact during home visits and in other settings, and by coordinating with appropriate team members as required.

**Conclusions:**

It is suggested that PHNs need to acquire practical skills depending on the characteristics of the case to cope with critical situations throughout the process of engagement.

**Supplementary Information:**

The online version contains supplementary material available at 10.1186/s12912-021-00706-z.

## Background

The activities of municipal public health nurses in Japan, who are in charge of a district and provide primary health care to all inhabitants there, begin with case exploration. The activities of public health nurses are characterized by home visits and necessary interventions when patients have health and safety issues, even if they do not request it. The cases in which public health nurses are involved are diverse and often involve ethical issues. Ethical issues refer to situations in which it is difficult to decide whether one’s actions as a professional are right or wrong, and which need to be examined. Typical examples of such situations are “intervention refusal”, “conflict between the patient and family”, “misunderstanding and conflict among neighbors”, and “necessary support beyond rules or guidelines” [1].

In a nationwide survey of public health nurses (PHNs) working in local governments in Japan, the number of those who had encountered “intervention refusal” was 395 (75.1%) out of 526 respondents, which was the highest number among the other nine types of issues [1]. In addition, the subjective level of difficulty for each issue (10 points for “very difficult” to 0 points for “not difficult at all”) was remarkably high for “intervention refusal”, with an average score of 7.3 points, compared to 6.7 to 5.8 points for the other nine items [1]. This suggests that it is necessary to first clarify the actual situation of cases of intervention refusal and the characteristics of support for them in order to provide smooth support without difficulty when PHNs encounter cases of intervention refusal in the future.

A CINAHL Plus search of articles from the past 10 years (2008–2018) for “TI. refusal AND (community health OR community care) NOT school refusal” yielded 4 hits, but the refusals included 2 vaccines, 1 condom, and 1 ministerial refusal. There were no papers that dealt with intervention refusal by PHNs or other professionals. The principles on which public health nurses have based their practice include Health for All in primary health care [2], a global health strategy, and “leave no one behind” in the 2030 Agenda adopted by the United Nations [3]. Nevertheless, the lack of findings on the characteristics of cases of refusal to intervene that may be left behind in the community and the support for them was considered to be a serious problem.

The purpose of this study is to clarify the actual situation of the cases and the characteristics of support, focusing on mothers and their children, and elderly persons among the cases of intervention refusal encountered by PHNs in Japan. The reason for this focus is that the initial involvement was by the PHN nurse alone in many of these cases, and we thought that many suggestions could be obtained for PHNs in developing primary health care.

In this paper, intervention refusal is defined as “a situation in which parties concerned (including the person and family) with problems related to life, the right to life, or livelihood refuse to accept intervention by other people, such as professionals or neighbors.

## Methods

This research was performed with full-time PHNs working for prefectural and municipal governments by an anonymous self-administered questionnaire. Questionnaires were sent by regular mail to supervising PHNs in 47 prefectural governments and 97 designated cities and special wards in which public health centers are established. The supervising PHNs were requested to distribute the questionnaires to 11 regions, which have different characteristics, in their jurisdictions.

The purpose of the questionnaire was to clarify the ethical issues encountered by PHNs and the actual state of support; the analysis of the quantitative data has already been published elsewhere [1]. This paper focuses on “intervention refusal”, one of the 10 ethical issues surveyed, and uses text data from one memorable case that PHNs had encountered, which they were asked to describe. We report in this paper the results of the analysis of the cases of mothers and their children, and elderly persons.

Respondents were asked to describe the following two matters set by the authors in original for this study (Supplementary file 1) regarding one memorable case of intervention refusal they had encountered: (1) specific situation about refusal of intervention (who refused what in which way); and (2) support provided to try to improve the situation (with whom and how PHNs were involved in the situation). Data were analyzed by the qualitative and descriptive analysis method. The characteristics of the cases and the support were categorized according to the situation of the case: the person who refused, the content of the refusal, the people and institutions involved in the support, and the content of the support provided, and the number of descriptions was summarized and interpreted in a table. The reliability of the classification was ensured by having two researchers create the classification criteria, then three researchers independently classified each of the mothers and their children, and the elderly persons, and then reviewed any discrepancies until the researchers were in agreement.

For ethical considerations, the purpose of the survey, protection of personal information, respected will of respondent candidates to refuse to participate in the survey, and any other relevant matters were explained in writing, and the questionnaire document included a tick box for expressing cooperation in the survey. Those who consented to do so checked it. The research plan was approved by the Ethics Committee for Observational Study, Osaka University Hospital (Approval No. 17302 dated December 26, 2017).

## Results

Out of 1584 questionnaires distributed, 534 were collected (response rate of 33.7%) and 526 were valid (valid response rate of 33.2%). Among the valid responses, 250 PHNs described relevant cases, of which 123 cases involved intervention refusal, 20 were mothers and their children, and 38 were elderly persons. The other 64 cases were adults, of which 49 (76.6%) were mentally challenged and the rest were mostly sick persons, and refusal of medical consultations and care services was the main reason for refusal.

The attributes of the 58 public health nurses who described the cases are 44 (75.9%) with 20 years or more of experience, 41 (70.7%) with job titles above the section chief level, and 38 affiliations with prefectures and cities with public health centers (65.5%), and the number of municipalities was 20 (34.5%).

1. Mothers and their children (Table 1)

**Table 1:** Cases of intervention refusal encountered by PHNs in Japan and characteristics of their support (mother/child)

			(1) With child abuse *n* = 8	Without abuse *n* = 12		*N* = 20
Described cases of intervention refusal				(2) With mental disorder	(3) Other	Total
		Total	8	7	5	20
**Refuser**		Mother	3	5	2	10
		Parents	4	1	3	8
		Male cohabiter	1	0	0	1
		Child (along with the mother in 1 case)	0	2	0	2
**Intervention refused (multiple answers)**	Administrative services	Home visit, etc.	3	2	2	7
		Advice to improve the nurturing environment	2	3	1	6
		Institutionalization	2	1	1	4
		Developmental support for the child	1	1	0	2
	Medical services	Recommendation of medical consultation or treatment	1	2	1	4
		Test for definite diagnosis	0	0	1	1
	Community support	Going to school	1	0	1	2
		Reporting of the missing child	1	0	0	1
**Person/ agency involved in support** **(multiple answers)**	Administrative agencies	PHN	8	7	5	20
		Other local government departments	3	5	3	11
		Child guidance center	6	3	2	11
		Child and family support center	3	1	1	5
		Public assistance worker	3	1	0	4
		Police	2	1	0	3
	Medical agencies	Medical institution	2	5	2	9
		Private midwife	0	1	1	2
	Health/welfare agencies	Welfare service office for the disabled	0	2	0	2
		Social welfare council	0	1	0	1
	Educational institutions	School	3	1	1	5
		Nursery school	1	1	1	3
	Relatives	Parents of the parents living outside the prefecture	1	0	0	1
	Inhabitants	Welfare worker	2	1	1	4
		Community consultant including mother-child consultant	2	0	0	2
**Category of support (multiple answers)**	Direct support	Continued contact	8	7	5	20
		Of which: Repeated home visits	4	1	2	7
		Childcare support	3	4	2	9
		Persuasion and reminder	3	1	1	5
		Relationship building	1	1	1	3
		Approach to the husband	1	1	0	2
		Childbirth support	1	1	0	2
		Livelihood support	1	0	0	1
		Job assistance	0	1	0	1
		Regular hospital visits	0	1	0	1
		Observation in collaboration with the community midwife	0	1	0	1
	Team approach	Collaboration with multiple professions	8	7	5	20
		Case conference	4	2	4	10
		Engagement of key person	1	1	0	2
		Approach from the midwife	0	1	0	1
	Use of social resources	Introduction of service	3	2	2	7
		After-school service for children	0	1	0	1
		Short stay of the mother	0	1	0	1

The 20 cases were divided into three categories: (1) 8 cases of abuse of children; (2) 7 cases without child abuse and with mental disorders; and (3) 5 other cases. In (1) through (3), the majority of refusals were by mother or parents (18 cases), and the others were by a man living with an abused child not related by blood, and two were by a child (one of which was by both mother and child).

The content of refusal was categorized into administrative services, medical services, and community support. In Category (1), there were 3 cases of refusal of home visits with the aim of confirming the safety of children and interviewing mothers and their children, and 2 cases of refusal of admission of children to child care facilities or nursery schools for the purpose of trying to separate mothers from their children. In one particular case in Category (1), advice to contact the police about a child who had run away from home and was missing was refused.

In 7 cases in Category (2), all mothers suffered from, or were suspected to be suffering from, some type of mental disorder. In 3 cases, advice to improve the nurturing environment, and in 2 cases, recommended visits to or treatment at mental clinics were refused.

The backgrounds of the 5 cases in Category (3) that did not have abuse or mental illness were characterized by individuality, although these are not shown in the table. They refused a variety of services including the following: mother or parents who stuck to their own methods of raising children; parents who refused testing to make a definite diagnosis of Down’s syndrome; and women who experienced unwanted pregnancy and gave birth without receiving any antenatal care.

Although some respondents did not sufficiently describe the people and agencies involved in their support, PHNs provided support in collaboration with an average of 3.2 (range 1–6) multi-disciplinary and multi-agencies in addition to themselves. The PHNs most frequently collaborated with other departments of the local government and child guidance centers, which were described in 11 cases (55.0%) each. The next most common organizations were, in order, medical institutions in 9 cases, child and family support centers and schools in 5 cases each, and public assistance workers and welfare commissioners in 4 cases each.

The support was categorized into direct support, team approach, and social resource utilization. The most common type of direct support was continuous contact, which was applicable to all of the respondents. Among these, repeated home visits were described in 7 cases. The contents of support included childcare support in 9 cases and persuasion and reminders in 5 cases. In the team approach, there was collaboration with multiple professions in all cases, and there were descriptions of case conferences in 10 cases. As for the use of social resources, 8 cases described the use of services, excluding duplication.

2. Elderly persons (Table 2)

**Table 2:** Cases of intervention refusal encountered by PHNs in Japan and characteristics of their support (elderly)

			(1) Self-neglect *n* = 27				(2) Abuse by a family member, etc. *n* = 11			*N* = 38
Described cases of intervention refusal			With dementia	With mental disorder	Other	Subtotal	With dementia	Other	Subtotal	Total
		Total	7	6	14	27	4	7	11	38
**Refuser**	The elderly	The elderly living alone	5	6	10	21			0	21
**(multiple answers)**		The elderly living with others	1		4	5	2	1	3	8
		Both husband and wife	1			1		1	1	2
	Cohabiters	Spouse				0	2	2	4	4
		Children and their family			1	1	3	2	5	6
		Any other cohabiter				0	1		1	1
	Relatives living separately					0		1	1	1
**Intervention refused (multiple answers)**	All human relations		1	1	5	7	1	3	4	11
	Administrative services	Visit, support, and advice from PHNs	2	3	2	7	3	3	6	13
		Involvement of public agencies	2		1	3	1		1	4
	Medical services	Treatment		2	4	6		2	2	8
		Consultation and house call	2	3	4	9			0	9
	Nursing care services	Use of nursing care services	1		5	6		3	3	9
		Recommendation of institutionalization			3	3	1		1	4
	Community support	Advice from neighbors	3	1		4		1	1	5
**Person/ agency involved in support (multiple answers)**	Administrative agencies	PHN	7	6	14	27	4	7	11	38
		Other local government departments	4	4	7	15	1	4	5	20
		Police	1	2	1	4		1	1	5
		Fire station/emergency services		1	1	2			0	2
		Family court				0	1		1	1
	Medical agencies	Medical institution	1	3	4	8		3	3	11
		Personal doctor or visiting physician	2	2	4	8	1	4	5	13
		Home-visit nursing (NS, PT, OT)			1	1		3	3	4
	Welfare-related organizations	Community general support center	3	2	7	12	3	1	4	16
		Care manager or home care support office	1	1	5	7	1	2	3	10
		Care facilities (including group homes and day service)	2	1	3	6	2		2	8
		Attendant service (caretaker)	1	1	2	4		2	2	6
		Other relevant organizations			3	3	2	1	3	6
		Social welfare council	1		3	4			0	4
	Rights protection-related	MSW, PSW		2	2	4		1	1	5
		Rights advocacy center		1	1	2			0	2
		Disability-related office		2		2			0	2
		Judicial scrivener				0		1	1	1
		Bank staff		1		1			0	1
	Relatives	Family member living separately	3	3	2	8		2	2	10
		Distant connection or relative	1	1	4	6		1	1	7
		Friend or religious group	1	1	2	4			0	4
	Inhabitants	Welfare worker	3	1	6	10	1	1	2	12
		Neighbor	2	2	2	6	2	2	4	10
		Landlord			2	2			0	2
		Head of neighborhood association			1	1			0	1
**Category of support (multiple answers)**	Direct support (daily life)	Home visit (individual or team)	5	5	13	23	4	7	11	34
		Arrangement of communication with family members or relatives living separately (telephone, letter, e-mail)	2	4	9	15	1	2	3	18
		Safety confirmation (visit in turns or meal delivery)	1	3	5	9		2	2	11
		Comprehensive observation or observation of daily life	3	2	3	8	1	1	2	10
		Information gathering (from neighbors or friends)	2	1	1	4	2		2	6
		Interview or attentive hearing	1		1	2		3	3	5
		Health check	1		1	2			0	2
		Support for end-of-life care			2	2			0	2
		Approach to family by letter following refusal of visit				0	1		1	1
		Ambush interview				0	1		1	1
	Direct support (treatment and institutionalization)	Hospitalization support (emergency transportation, pickup or persuasion)	2	4	6	12		2	2	14
		Support for medical consultation or home visit	2	4	5	11		2	2	13
		Compulsory measure or protection	2		1	3	2		2	5
		Support or arrangement for institutionalization		1	2	3	2		2	5
	Team approach	Team support through information exchange and collaboration	6	4	10	20	3	5	8	28
		Consultation and arrangement for contact with doctor	2	2	4	8	1	1	2	10
		Contact with welfare worker, etc.	2	1	3	6		1	1	7
		Ongoing care conference	2	1		3	1	1	2	5
		Contact with police or fire station	1	1	1	3			0	3
	Use of social resources	Arrangement for, or introduction of home care service	3	1	3	7	2	4	6	13
		Application for long-term care insurance benefit	3	1	3	7			0	7
		Introduction of caretaker	2	1	1	4		2	2	6
		Arrangement with personal doctor	1		1	2		3	3	5
		Introduction of home-visit nursing care			1	1		3	3	4
		Application for welfare benefit			2	2			0	2
	Rights protection-related	Respect for personal preference or intention	1	1	1	3			0	3
		Financial management (in coordination with relevant institution)		2		2		1	1	3
		Action to address accumulation of garbage at home	2		1	3			0	3
		Procedure or arrangement for selecting guardian				0	2		2	2
		Family court				0	1		1	1
		Support for will writing (not in time)		1		1			0	1
		Action to address too many pets			1	1			0	1

The 38 cases were divided into two categories: (1) 27 cases of self-neglect, and (2) 11 cases of abuse by spouses, children, and cohabiters. In Category (1), 21 (77.8%) were living alone. In Category (2), neglect was noted in 10 cases and in four of them, financial exploitation was found, although these are not shown in the table.

Those who refused, regardless of whether they lived alone or with others, were themselves in 31 cases, accounting for 81.6%. When interventions were refused by spouses or relatives, they may have been wrong in assuming that such support interventions were not required, or issues of property inheritance were involved in the refusal. Among 38 cases, dementia was found in 11 cases (28.9%), mental disorders in 6 cases (15.8%), and 11 (28.9%) had other serious illnesses such as intractable diseases.

As in the case of mothers and children, refusals included administrative services, medical services, and community support, but in the case of the elderly, refusals were categorized as refusal of all human relations and refusal of care services, each of which was found in 11 actual cases. The most common reasons for both (1) and (2) were visits, support, and advice from PHNs (together 13 cases, 34.2%) and use of nursing care services (together 9 cases, 23.7%). In (1), refusal to receive medical care services such as treatment, consultation, and house calls was particularly common, with 14 respondents (51.9%).

Regarding the people and institutions involved in the support, PHNs provided support in collaboration with an average of 4.3 (range: 1–9) multi-disciplinary and multi-agencies in addition to themselves. The organizations with which the public health nurses collaborated most often were health and welfare related organizations, with 30 actual cases (78.9%), and among them, with community general support centers in 16 cases (42.1%). This was followed by medical institutions in 21 cases (55.3%) and other departments of local governments in 20 cases (52.6%). Characteristically, there was involvement of rights advocacy (MSW/PSW in 5 cases, rights advocacy center in 2 cases, etc.), and inhabitants (welfare committee members in 12 cases, neighbors in 10 cases, etc.) were also frequently involved.

In terms of the contents of support, direct support for daily life was the most diverse and numerous, followed by direct support for medical treatment and admission, team approach, use of social resources, and various contents classified as rights protection-related. In regard to direct support, home visits were described most frequently, in 34 cases (89.5%). Although it could not be shown in the Table 2, PHNs tried to contact the persons concerned by various methods, such as leaving a message at home, asking key persons or commissioned welfare volunteers for cooperation, or visiting homes together with welfare workers, until they started to make their interventions accepted in these cases. For monitoring activities and handling critical and life-threatening situations, PHNs collaborated with persons engaged in various duties, depending on the situation, and sometimes worked with the police when they were taking legal action.

## Discussion

The results revealed that except for one case, interventions involving mothers and children were refused by mother or parents. The refusals were related to child abuse, parental mental illness, obsessiveness, and complex backgrounds. It was suggested that PHNs need to acquire practical skills to determine the extent of urgency of separating mothers from their children on the basis of comprehensive assessment, and to identify methods of adequately approaching the parents and children concerned in cooperation with multiple organizations engaged in collaborative partnership through case study meetings.

The results revealed the actual status of intervention refusal in elderly persons, that is, interventions are frequently refused by elderly persons themselves in the case of self-neglect and by family members living with the elderly in the case of abuse. The refusals were related to mental disorders or dementia and living alone. It is suggested that PHNs need to acquire practical skills with which, depending on the characteristics of the case including disease, history, and its impact, they can conduct monitoring activities and take effective actions at the optimal timing to cope with critical situations throughout the process of engagement.

In both maternal and elderly cases, attempts were made to alleviate the situation of refusal to intervene, from detecting cases through contact during home visits and in other settings, and by coordinating with appropriate team members as required. Backhouse et al. [4], who conducted a systematic review of the strategies and interventions to reduce or manage refusals in personal care in dementia, found that “reducing elderspeak and negative communications, and some psychosocial interventions can reduce refusal behaviours in dementia.” In addition, Corvola et al. [5] analyzed four focus groups of case managers and users on refusal of care faced by case managers from elderly persons in complex situations, and recommended “refusal of care often is the result of the will of preserving one’s identity, compromised by illness. Individuals seek control on their life.”, and “To recognise an elderly person that refuses care as a unique individual who can make choices, secure his identity, and allow him to change.” By promoting the incorporation of these research findings, PHNs may be able to overcome the difficult situation of intervention refusal. From the perspective of “leaving no one behind,” it is necessary to consider the utilization of existing resources. In recent years, the application of implementation science to underserved populations from the perspective of equity correction has been considered in implementation science that has a variety of models [6]. In addition, the WHO has developed and provided various support tools to countries so that they can operationalize health plans to achieve the goal of “leaving no one behind” [7]. They may be useful in identifying who is vulnerable to being left behind, and in supporting the strengthening of national plans, health systems, or health programs, and thus in considering systematic measures to respond to cases of refusal of intervention.

In any case, more in-depth interviews with PHNs will be necessary to collect and accumulate information on the individuality of each case and effective approaches for various situations, and to present best practices.

### Limitations

Since the method of this survey was to ask participants to describe a case they had experienced, there were individual differences in writing style (sentences or bullet points) and content (specific or brief words only), and there were limitations in the analysis of data that were less rich than data collected through interviews. Another limitation is that the results of this survey are not rigorous in terms of clarifying the actual situation, as they are a summary of the participants’ descriptions of one case that made a strong impression on them, and not a survey of all the cases they encountered.

## Conclusions

- The characteristics of the cases and the support which the cases of intervention refusal encountered by PHNs in Japan were categorized according to the situation of the case, and the number of descriptions was summarized and interpreted.
- Most intervention refusals involving mothers and their children were by their mother or parents, and were more common in cases of child abuse and cases with parental mental illness.
- Intervention refusals involving the elderly were mostly by the elderly themselves in cases of self-neglect, or by family members living with them in cases of abuse, and the refusals were related to mental disorders, dementia, or living alone.
- In both cases, PHNs attempted to alleviate the situation of intervention refusal by finding out about the case through home visits and other contacts, and then coordinating with appropriate multiple professionals and communities as needed.
- From now on PHNs need to acquire practical skills with depending on the characteristics of the case to cope with critical situations, and ensure primary health care that contributes to “leaving no one behind.”

## Supplementary Information

**Additional file 1.**

## Data Availability

Data sharing is not applicable to this article as no datasets were analysed during the current study.

## Abbreviations

PHNs: Public health nurses
WHO: World health organization
