# Supplementary material for: Cases of intervention refusal encountered by public health nurses in Japan and characteristics of their support– qualitative analysis of described mother-child and elderly cases
Source: BMC Nurs. 2022 Feb 3;21:39. doi: 10.1186/s12912-021-00706-z (PMC8812212; doi:10.1186/s12912-021-00706-z)
Supplement: Supplementary file 1 — Additional file 1. [file 12912_2021_706_MOESM1_ESM.pdf]

Questionnaire on the ethical issues encountered by public health nursing and how to deal with them

Consent Box

☐ ☚ **If you consent to cooperate in our research, please tick the box on the left ☑ .**

1. In the following ethical issues in public health nursing (situations where you have trouble making decisions about issues related to life, the right to life and daily liveing), have you had any experience as a public health nurse to improve the situation?
Please mark ◯ on either Yes or No.

1	Conflict between the patient and his/her family	**Yes**	**No**
2	Intervention refusal	**Yes**	**No**
3	Limitation of the respect of the patient's will	**Yes**	**No**
4	Misunderstanding and conflict among neighborhoods	**Yes**	**No**
5	Protection of personal information	**Yes**	**No**
6	Inequality and disparity	**Yes**	**No**
7	Necessary support beyond rule or guideline	**Yes**	**No**
8	Necessary support beyond law and policy	**Yes**	**No**
9	lack of practical competency	**Yes**	**No**
10	limitation of responsibility	**Yes**	**No**

2. Please describe the following two matters regarding one memorable case above you had encountered:
(1) specific situation about refusal of intervention (who refused what in which way); and
(2) support provided to try to improve the situation (with whom and how PHNs were involved in the situation).

Please write first what number of ethical issues 1-10 corresponds to ➡ Number (   )

(1)

(2)
